# Impact of social support on the outcomes of occupational injuries in the construction sector

**DOI:** 10.1192/j.eurpsy.2023.2023

**Published:** 2023-07-19

**Authors:** I. Sellami, A. Hrairi, A. Haddar, N. Kotti, M. L. Masmoudi, K. Jmal Hammami, M. Hajjaji

**Affiliations:** occupational medecine, Hedi Chaker Hospital, University of Sfax; 2 occupational medecine, University of Sfax, Sfax, Tunisia

## Abstract

**Introduction:**

Occupational injuries in the construction sector constitute an important health problem affecting workers in their most productive years. The professional environment influences the outcomes of these accidents. The impact of social support among this vulnerable population may explain the difference in terms of outcomes of occupational injury.

**Objectives:**

Evaluating the impact of social support in occupational injuries’ outcomes among construction workers.

**Methods:**

A cross-sectional analysis was conducted during 9 months among construction sector workers victim of an occupational injury consulting for an Impairment Rating Evaluation and working. Socio-professional data and the accident’ outcomes were collected. Social support was evaluated by the Social Support Scale. The pain was evaluated by a Visual Analogue Scale.

**Results:**

Out of 51 injured workers, 96.1% were male. The mean age was 43.66 **±** 10.79 years. The majority of accidents took place in 2020 (49%). Upper arm injuries represented 41.2% of injured sites. The mean pain scale was 6.98±1.69 and the mean length of absence was 227.88 days±292.23. A proportion of 9.8 % had Low social support. Twenty-three subjects (45.1%) had not returned to work. Low social support was associated with a perception of stigma and discrimination (p=0.000), negative outlook of the future (p=0.003), low job satisfaction (p=0.000) and non-return to work (p=0.009). No association was found with pain, length of absence and sleep disorders.

**Conclusions:**

Social support may influence occupational injury outcomes. This finding highlights the need for further examination of social factors among this vulnerable population.

**Disclosure of Interest:**

None Declared

